# A Novel Framework for the Identification and Analysis of Duplicons between Human and Chimpanzee

**DOI:** 10.1155/2013/264532

**Published:** 2013-08-01

**Authors:** Trees-Juen Chuang, Shian-Zu Wu, Yao-Ting Huang

**Affiliations:** ^1^Genomics Research Center, Academia Sinica, Taipei, Taiwan; ^2^Department of Computer Science and Information Engineering, National Chung Cheng University, No.168 University Road Chiayi, Taiwan

## Abstract

Human and other primate genomes consist of many segmental duplications (SDs) due to fixation of copy number variations (CNVs). Structure of these duplications within the human genome has been shown to be a complex mosaic composed of juxtaposed subunits (called duplicons). These duplicons are difficult to be uncovered from the mosaic repeat structure. In addition, the distribution and evolution of duplicons among primates are still poorly investigated. In this paper, we develop a statistical framework for discovering duplicons via integration of a Hidden Markov Model (HMM) and a permutation test. Our comparative analysis indicates that the mosaic structure of duplicons is common in CNV/SD regions of both human and chimpanzee genomes, and a subset of core duplicons is shared by the majority of CNVs/SDs. Phylogenetic analyses using duplicons suggested that most CNVs/SDs share common duplication ancestry. Many human/chimpanzee duplicons flank both ends of CNVs, which may be hotspots of nonallelic homologous recombination.

## 1. Introduction

Human genome and other primate genomes consist of many repetitive sequences. Many of these are hotspots for nonallelic homologous recombination (NAHR) [1] or genomic rearrangements. Current estimates suggest that approximately 4%–6% of our human genome is composed of segmental duplication (SD) [1–3]. SD is a DNA segment ≥1 kb in size that occurs greater than once within the genome and typically shares ≥90% sequence identity [1, 4]. Genomic regions of SDs have been shown to be hotspots of copy number variations (CNVs), which is a DNA segment 1 kb or larger in size and presents different number of copies in the population. A number of SDs and CNVs have been known to highly associate with several complex diseases such as HIV-1 infection, glomerulonephritis, Parkinson, and Alzheimer diseases [5–8].

The completion of several sequencing projects provided abundant resources for mapping SDs in mammalian genomes. SDs are usually identified by self-comparison of the entire genome or by coverage analysis of overcollapsed shotgun sequences [2, 9]. For example, a genome-wide map of chimpanzee SDs was built by self-comparison of chimpanzee assembly and alignment of shotgun sequences to the human genome [10]. Through comparison of clone-ordered assemblies of human and mouse, She et al. [11] found that the amount of mouse SDs is comparable to that of human SDs. Recently, with the advent of array comparative genomic hybridization (aCGH), numerous CNVs have been discovered in several mammalian populations [12–14]. For example, Redon et al. [15] identified a total of 1,447 CNVs from 270 individuals across four populations, covering 360 megabases of the human genome. Perry et al. [16, 17] characterized a map of CNVs in chimpanzees and found that human and chimpanzee CNVs occur in orthologous regions far more than expected.

A number of statistical and combinatorial methods have been developed to identify SDs/CNVs on the basis of comparative genomics, microarray, or high-throughput sequencing platforms. For instance, comparative approaches aim to uncover genomic sequences with high similarity from whole-genome sequence alignment [3, 10, 11]. Computational methods on top of microarray platforms often identify genomic regions with high density of unusual intensity signals [18, 19]. On the other hand, algorithms for high-throughput sequencing platforms search for genomic segments with ultrahigh/low read depth or aberrant mapping distances [20].

Even though many duplications have been discovered and studied in the last decade, the underlying mechanism leading to these large duplications is still not well understood. To date, NAHR and retrotransposition are two mechanisms known to support many duplication events. NAHR, also termed ectopic recombination or unequal crossover, is a recombination error during meiosis in which the exchanged chromosomes were misaligned, leading to gain or loss of DNA segments [1, 21, 22]. The misalignment of NAHR has been suspected due to repetitive elements widespread in the genome. On the other hand, the activation of retrotransposons, retrovirus, and endogenous retrovirus (ERV) may also mediate retrotransposition of a few genes via reversely transcribing RNAs into DNAs and inserting them back to the genome [23].

**Figure 1:**
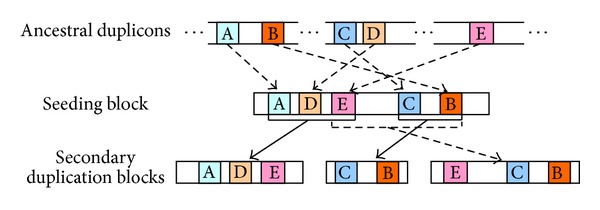
Ancestral duplicons are first aggregated into one seeding block that subsequently produces secondary duplication blocks.

In recent years, a few studies started to investigate the sequence composition within large duplications and found that the structure is a complex mosaic composed of smaller subunits called * duplicons* (with a minimum length of 100 bp) [2, 24, 25]. A two-step model has been established to explain this mosaic structure [26, 27] (see Figure 1). In this model, ancestral duplicons are first transposed and aggregated into one seeding block, which subsequently produces secondary duplication blocks. Duplicons within this complex mosaic cannot be readily uncovered by conventional multiple sequence alignment approaches. Thus, Pevzner et al. [28] developed an *A*-Bruijn graph algorithm for identifying duplicons from this mosaic structure. The *A*-Bruijn graph algorithm was then revised to discover 4,692 ancestral duplicons using human SDs and outgroup mammalian genomes [24]. Subsequently, Jiang et al. [9] compiled a library of known duplicon sequences and used this library to efficiently annotate SDs in a new genome.

The discovery of duplicons was based on comparing sequences of known SDs. In reality, due to the difficulty of assembling shotgun sequences in duplicated regions, large (>15 kb) and highly identical (>95%) SDs are often collapsed [11]. Furthermore, because these shotgun sequences are collected from only a few individuals in the population, SDs of unsampled individuals would be missed in the assembled genome [17]. Thus, a substantial amount of duplicons can be lost. In fact, CNVs have been viewed as a drifting and polymorphic form of SDs, and both are probably mediated by similar mechanisms [29]. A few studies have reported that only ~24% of CNVs are overlapped with SDs [15, 22], implying that CNVs may serve as alternative repository of duplicons. Recently, analysis of a fosmid clone indicated that a large segment of CNV is deleted owing to NAHR mediated by flanking duplicons [9]. However, the distribution of duplicons within CNVs and their mosaic structures in human and other primates remains poorly investigated.

In this paper, we develop a Hidden Markov Model (HMM) for efficiently annotating duplicons within CNVs and assess the statistical significance of each duplicon. Our results indicate that the mosaic structure composed of duplicons is common in CNVs and SDs of both human and chimpanzee. Although our duplicons are annotated from a subset of CNVs, other CNV regions are found to have significantly higher density of these duplicons. Phylogenetic analyses suggest that many CNVs/SDs share common duplicons and ancestry, and these CNVs/SDs are usually centered around a few core duplicons shared by majority of duplications with common ancestry. In addition, a number of duplicons are found to flank both ends of human and chimpanzee CNVs, creating hotspots of nonallelic homologous recombination. Compared with previous functional analysis on CNVs, these duplicons are also enriched for regulation of immune process and response to stimulus but underrepresented in cell adhesion.

## 2. Method

### 2.1. Data Preprocess and Problem Formulation

**Figure 2:**
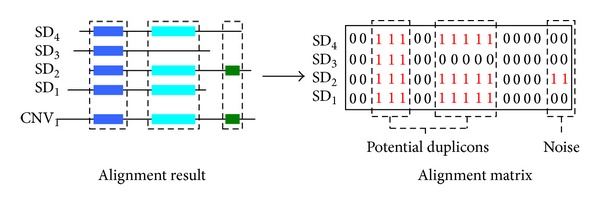
The left figure illustrates one alignment result. Fragments with the same color represent the subsequences on CNV_1_ and SDs having high similarity. The right figure illustrates the alignment matrix corresponding to the alignment result. In this matrix, the two clusters of 1s are potential duplicons, whereas the remaining parts are probably noise.

We downloaded a total of 50,339 human SDs from the University of California Santa Cruz genome browser (http://www.genome.ucsc.edu) [2]. 1,447 human CNVs screened by a tiling array and an SNP genotyping array are obtained from Redon et al. [15]. We used Megablast [30, 31] to align all SDs against each CNV (The parameters of Megablast are set as follows: −e 0.0001, −F F, −W 34, and −M 1000000). We found that megablast is able to complete the alignment task under this setting within one week, whereas the regular blastn is unable to finish within a reasonable period of time. Although the speed can be theoretically improved by using word size larger than 34 bp, we did not observe significant differences when further enlarging the word size. According to the alignment result, we construct an “alignment matrix” for each CNV (Figure 2). Denote *n*_*k*_ as the length of the *k*th CNV sequence and *m* as the number of SDs which can be aligned to the *k*th CNV. Let *A*_*k*_ = (*a*_*ij*_) be a binary *m* × *n*_*k*_ matrix. Each element in the matrix *A*_*k*_ is defined as *a*_*ij*_ = 1 if the *i*th SD is aligned to the *j*th position of the *k*th CNV and *a*_*ij*_ = 0 otherwise, where 1 ≤ *i* ≤ *m* and 1 ≤ *j* ≤ *n*_*k*_. Note that gaps and mismatches are excluded in *A*_*k*_. Theoretically, real duplicons tend to produce segments of consecutive “1s” with higher frequency and longer length in the matrix. On the other hand, segments of 1s due to random or occasional alignments are less frequent and relatively shorter. In the following, we describe an HMM for identifying duplicon regions with sufficient frequency and length.

### 2.2. Hidden Markov Model

The HMM is specified by five sets of parameters, *λ* = (*S*, *O*, *π*, *T*, *E*), where *S* is the set of states, *O* is the set of observation, *π* is the initial state, *T* is the set of state transition probabilities, and *E* is the set of emission probabilities. We define *S* = (*D*_1_, *D*_2_, *N*) as our state alphabet set, where *D*_1_ and *D*_2_ represent two duplicon states, and *N* is the nonduplicon state. We use two duplicon states in order for distinguishing adjacent duplicons. Our HMM starts at the initial state *π* with equal transition probability to one duplicon state and the nonduplicon state.

**Figure 3:**
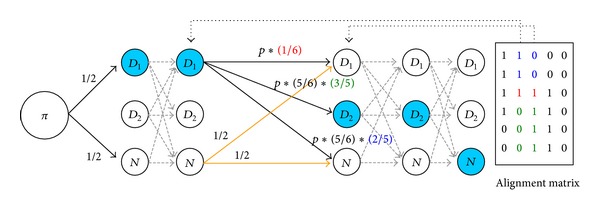
An example of state transition probability of our HMM. We take the second and third columns as an instance and highlight the transition probability for *D*_1_ state. Note that *ω* = 1/6 and *γ* = 2/5. The expected Viterbi path in this instance is *D*_1_, *D*_1_, *D*_2_, *D*_2_, *N*.

In our HMM, the state transition probabilities *T* are designed to approximate the length of known duplicons and reflect the transition likelihood implied by 0/1 patterns of two adjacent columns in the matrix. First, the average length of known duplicons *L* is computed from the duplicon library [9]. The probability of transition from one duplicon state to itself (e.g., *D*_1_ to *D*_1_) is set to *p* = 1 − 1/*L*, which corresponds to a geometric distribution with mean *L*. In addition, we also compute the frequencies of three 0/1 patterns (*f*_1,1_, *f*_0,1_, and *f*_1,0_) in two adjacent columns. For example (see Figure 3), *f*_1,1_, *f*_0,1_, and *f*_1,0_ in the first two columns of the matrix are 3, 0, and 1, respectively. Intuitively, *f*_1,1_, *f*_0,1_, and *f*_1,0_ imply the likelihood of transition to the same duplicon state, the other duplicon state, or nonduplicon state, respectively.

Let *ω* = *f*_1,1_/(*f*_1,1_ + *f*_0,1_ + *f*_1,0_) and *γ* = *f*_0,1_/(*f*_0,1_ + *f*_1,0_). For each duplicon state, we define three state transition probabilities: (1) transition to the same duplicon state with probability *pω*; (2) transition to the other duplicon state with probability (1 − *pω*)*γ*; (3) transition to nonduplicon state with probability (1 − *pω*)(1 − *γ*). The transition probability for the nonduplicon state is set to be equally likely. Figure 3 illustrates an example of our state transition probabilities.

Theoretically, the columns of a real duplicon should have higher frequency of 1s than those of nonduplicon columns. Thus, we define observation *O* = (*o*_1_, *o*_2_,…, *o*_*n*_*k*__) as the number of 1s in each of the *n*_*k*_ columns, respectively. The emission probability *E* of the *i*th duplicon state is designed to reflect the probability of observing *o*_*i*_ 1s, assuming that this position is a real duplicon. First, we estimate the probability of observing a duplicon in one SD from the known duplicon library [9]. That is, *P*_*o*_ = *C*/*M*, where *C* is the average copy number of one duplicon and *M* is the number of total SDs in the duplicon library. Let *k* be the number of 1s in the column and *n* the number of SDs in the alignment matrix. The emission probability on the duplicon state is defined as *P*_*d*_ = ∑_*i*=0_^*k*^(_*i*_^*n*^)*P*_*o*_^*i*^(1 − *P*_*o*_)^*n*−*i*^, corresponding to a cumulative binomial distribution. And the emission probability on nonduplicon state is defined as 1 − *P*_*d*_.

The maximum probability path in the HMM starting from *π* and ending at state *S*_*o*_*n*_*k*___[*x*] is given by

(1) P(V ∣ Ak,λ)=P(S[x] ∣ π) ×P(So1[x])∏i=2nkP(Soi[x] ∣ Soi−1[x]) ×P(Soi[x]).

This maximum probability path is found by the Viterbi algorithm [32], and all positions are assigned to one of the three states. We identify segments with at least 100 *D*_1_ or *D*_2_ duplicon states as potential duplicons.

### 2.3. Permutation Test

**Figure 4:**
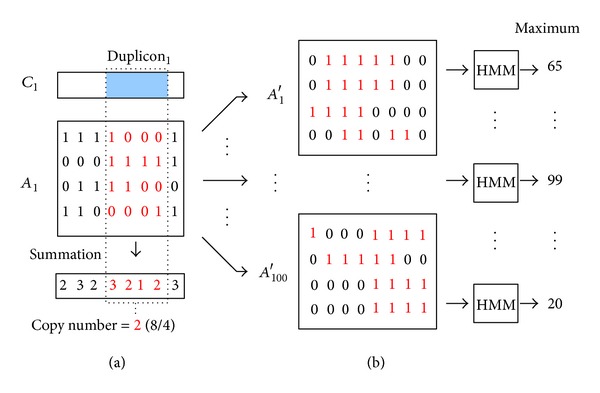
(a) An example of computing copy number for a duplicon. The average copy number of duplicon_1_ is 8/4 = 2. (b) Flow of permutation test. Each consecutive 1s in *A*_1_ is randomly relocated to create 100 artificial alignment matrices *A*_1_′, *A*_2_′,…, *A*_100_′.

The statistical significance of each potential duplicon is assessed by a permutation test. We define “copy number” of a duplicon as the average number of SDs aligned to each position of the duplicon (Figure 4(a)). The permutation test computes the probability of observing the copy number of a potential duplicon from permutated data. Real duplicons tend to have sufficient number of copies, which are less likely to be observed by chance only. In the permutation test, each segment of consecutive 1s in the alignment matrix is randomly relocated to create an artificial matrix (Figure 4(b)). 100 artificial matrices are created separately for each alignment matrix. Then, duplicons of each artificial matrix are identified by applying our HMM. The maximum copy number among all duplicons in each artificial matrix is recorded. For each potential duplicon of the original matrix, the *P* value is defined as the fraction of artificial matrices for which maximum copy number is larger than that of the potential duplicon. Only those duplicons with *P* value <0.01 are retained as our final solution.

For instance, suppose we have 30 copies of a potential duplicon observed in alignment matrix *A*_1_. After permutation test, there are ten maximum copy numbers (from artificial simulations) greater than 30 (*P* value = 0.1 > 0.01). This potential duplicon would be eliminated due to its nonsignificant *P* value. On the contrary, if there is no maximum copy number of artificial duplicons in *A*_1_ greater than 30, the duplicon (*P*  value = 0 < 0.01) is assessed as a potential true duplicon.

### 2.4. Gene Ontology Analysis

We retrieve known genes annotated by Ensembl (http://www.ensembl.org). Duplicons overlapped with these known genes are included in our analysis. In order to investigate the functional bias of these duplicons, we identified over- and underrepresented functions defined by gene ontology (GO) term analysis (http://www.geneontology.org). For each GO subcategory (level 2 and level 3) of biological process, cellular component, and molecular function, we compute the numbers of all genes and all duplicons that fall into each subcategory. The statistical significance of over- or underrepresentation in any GO subcategory is computed by chi-square test. *P* values are corrected using Bonferroni correction for multiple testing. The subcategories with *P* < 0.05 are investigated in our analysis.

### 2.5. Hierarchical Clustering and Phylogenetic Analysis of Duplicons

A binary “phylogenetic profile” was constructed based on the extent of shared duplicons for each duplication segment composed of ten or more duplicons. The duplication segment is defined as the chimpanzee SDs and CNVs (chimpanzee specific, human specific, and human/chimpanzee shared) in which the segments are aligned by our duplicons with sequence identities ≥95% and length ≥100 bp. If a duplicon is present within a duplication segment, we assigned “1” for that duplicon in duplication segment, otherwise assigned “0,” generating a binary phylogenetic profile for each duplication segment. If there is no shared duplicon among two duplication segments, these two segments are considered to have no related evolutionary history. A duplication group is a cluster of duplication segments grouped based on the amount of shared duplicons. Complex duplication segments were then clustered into several duplication groups by hierarchical clustering on the basis of the similarity of their phylogenetic profiles. ClustalW is used to generate phylogenetic clusters of these profiles (http://www.ebi.ac.uk/Tools/clustalw2/index.html). Each clade in the phylogenetic tree stands for a duplication group in our analysis.

## 3. Results and Discussion

### 3.1. Novel Duplicons Annotated by Our Pipeline

**Figure 5:**
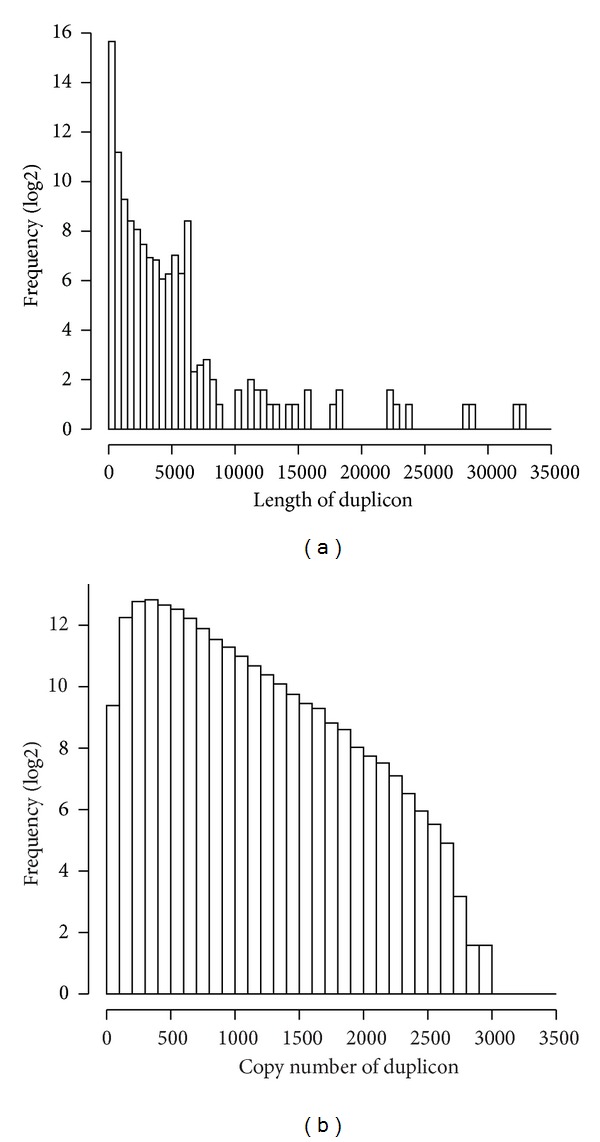
(a) The distribution of lengths of our duplicons. (b) The distribution of copy numbers of our duplicons.

The binary and source code of the entire pipeline have been encapsulated via bash script and are available at http://www.cs.ccu.edu.tw/~ythuang/Tool/HMMDupFinder/. We downloaded a total of 50,339 human SDs from the University of California Santa Cruz genome browser (http://www.genome.ucsc.edu) [2]. 1,447 human CNVs screened by a tiling array and an SNP genotyping array are obtained from Redon et al. [15]. We used Megablast [30, 31] to align all SDs against each CNV and created 1,447 alignment matrices (see Section 2). We design and implement a HMM and run the HMM on alignment matrices for annotating duplicons. A total of 102,405 initial duplicons were found by the HMM. After filteration by a permutation test (*P* < 0.01) and removal of identical duplicons, 56,377 unique duplicons were retained. These duplicons are spread among 1,095 CNVs. On average, each CNV contains approximately 54 unique duplicons. There are 963 CNVs (88%) having two or more identical duplicons within the genomic region, and 2,994 duplicons appear twice or more in the same CNV. ~71% of our duplicons are novel compared with known duplicons in [9].  Table 1 lists numbers of duplicons on each chromosome.  Figure 5 illustrates the distribution of length and copy number of all duplicons. The average length of our duplicons is 425 bp, which is shorter than that of duplicons annotated by *A*-Bruijn graph method (~4,651 bp) [9, 24]. This is because *A*-Bruijn graph methods chain duplicons in proximity or across repeats, whereas our HMM will distinguish adjacent duplicons (see Method). On the other hand, the average copy number of our duplicons is 644, which is much larger than that of previous study (~6 copies) [24]. This is not unexpected since our method assessed the statistical significance of each duplicon by a permutation test on the copy number. Therefore, duplicons without sufficient copy number are discarded. Nevertheless, even with a more stringent criterion, we still identified many duplicons with long length (>10,000 bp) and with high frequency of copies (>2,000 copies).

### 3.2. Mosaic Structure is Common in Human and Chimpanzee

Our duplicons were annotated by CNVs and SDs in human. The distribution of these duplicons within CNVs and SDs in other primates is still unclear. Therefore, we downloaded chimpanzee and human SDs identified by self-comparison of the chimpanzee assembly and alignment of shotgun sequences [10]. These SDs were classified into three categories: 219 chimpanzee specific SDs (i.e., chimpanzee SDs that do not overlap with any human SDs), 618 human specific SDs (i.e., human SDs that do not overlap with any chimpanzee SDs), and 658 human/chimpanzee shared SDs. Our duplicons were BLAST aligned to SDs.  Table 2 lists the number (and percentage) for each type of SDs containing our duplicons. The results indicated that our duplicons also appeared in majority of chimpanzee specific SDs (which are not included in our annotation process). In fact, over 98% of SDs in all three categories contained our duplicons. Furthermore, each SD includes an average of 24~43 duplicons, regardless of chimpanzee specific or human specific SDs. Consequently, these results suggest that the mosaic structure composed of duplicons is not only limited to human SDs but is also common in chimpanzee SDs.

Similarly, we compare the distribution of duplicons within CNVs between human and chimpanzee. 353 and 438 CNVs in the genomes of 30 humans and 30 chimpanzees were obtained from Perry et al. [17], respectively. These CNVs were also classified into 288 chimpanzee specific CNVs, 207 human specific CNVs, and 296 human/chimpanzee shared CNVs. As shown in Table 2, all of chimpanzee specific CNVs also contain our duplicons, indicating that these duplicons are not limited to human CNVs. Overall, the majority of CNVs in three categories includes our duplicons, and each CNV contains approximately 16~22 duplicons. This phenomenon shows that duplicons are also common in chimpanzee CNVs. Compared with the results on SDs, the average numbers of duplicons on each CNV or SD are also quite similar. Consequently, the mosaic structure of juxtaposed duplicons may be common within SDs and CNVs in hominoid.

### 3.3. Phylogenetic Analysis and Identification of Core Duplicons

**Figure 6:**
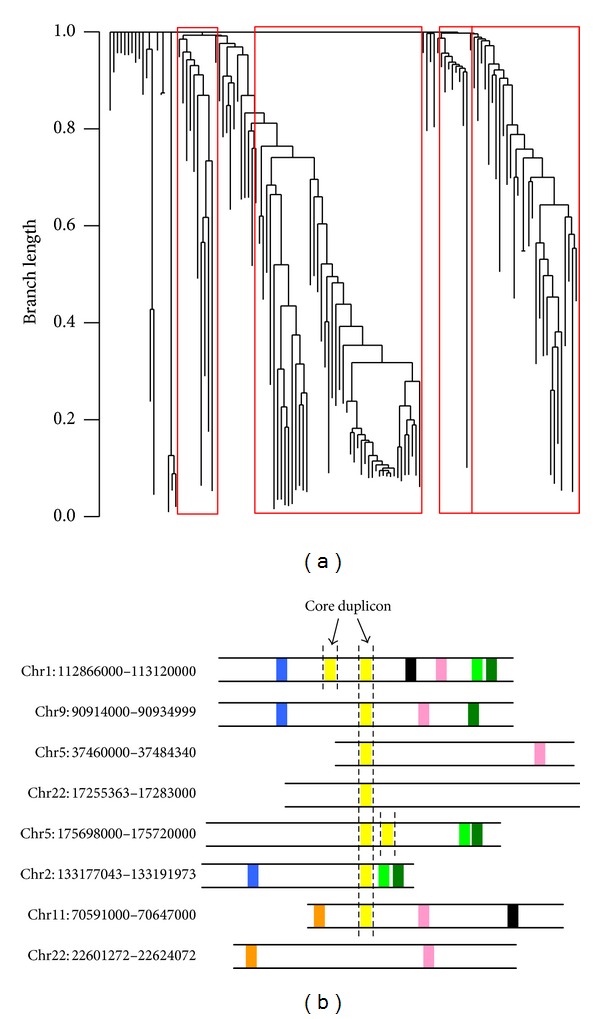
(a) Chimpanzee specific SDs are clustered by running Neighbor-Joining algorithm on their phylogenetic profiles constructed by duplicons. Four clades are revealed in this phylogenetic tree. (a) A cluster of chimpanzee specific SDs with shared duplicons. Different colors denote distinct duplicons. A core duplicon shared by a majority of these SDs is highlighted by vertical dash lines.

A number of studies suggested that secondary duplications may have occurred recently among existing duplications, and these recent duplications tend to share more duplicons in common [24]. Thus, we reconstruct phylogenetic history of these SDs and CNVs using a representation of duplicons called phylogenetic profile [24]. A phylogenetic profile is created for each SD and CNV based on the presence or absence of each duplicon (see Method). For each group of human specific, chimpanzee specific, and human/chimpanzee shared SDs and CNVs from [17], a phylogenetic tree is reconstructed by running the Neighbor-Joining algorithm on their phylogenetic profiles constructed by duplicons [33]. That is, the branch length reflects the degree of SDs/CNVs having the same duplicons in common.  Figure 6(a) illustrates one phylogenetic tree reconstructed via duplicon profiles for chimpanzee specific SDs, where the other phylogenetic results can be found in supplementary Figures 1–6 (see Supplementary Material available online at http://dx.doi.org/10.1155/2013/264532). Together, these results suggested that many of these SDs and CNVs share common ancestry of duplications, which are probably owing to recurrent duplications from a few seeding duplication blocks.

A large fraction of recent duplications have been shown to be centered around a small subset of “core duplicons” [24]. The structure of core duplicons with flanking duplicons is speculated to drive the rapid expansion of SDs widespread in hominoid genomes. The phylogenetic clustering of SDs or CNVs with common ancestry can be further used for identifying these core duplicons, which are shared by majority of SDs/CNVs in the same clade. A core duplicon is defined as a duplicon shared by >67% of SDs/CNVs in the same clade [24].  Figure 6(b) illustrates one core duplicon found in a clade. A total of 639 core duplicons were found. In summary, our analysis shows that many SDs and CNVs in human and chimpanzee have a nonrandom clustering structure of common duplicons and ancestry, and a number of core duplicons with flanking duplicons may trigger further duplications leading to novel SDs or CNVs.

### 3.4. Comparison of Duplicon Densities in CNVs and Non-CNV Regions

Duplicons identified by our pipeline were based on a subset of known CNVs in the human genome. As novel CNVs were reported by new sequencing projects, the power of our method can be estimated by observing the density of our duplicons in other newly annotated CNVs and non-CNV regions. Coordinates of 21,678 human CNVs are obtained from the Database of Genomic Variants (http://projects.tcag.ca/variation). Overlapping CNVs are merged and the 1,447 training CNVs used for annotating our duplicons are excluded. Non-CNV regions are defined as the genomic regions in between these known CNV regions. Note that non-CNV regions may still contain some CNVs not annotated. We first align all duplicons against the entire human genome and compute the duplicon density in CNV and non-CNV regions. Since core duplicons tend to be shared by more CNVs than noncore duplicons, each duplicon is assigned a weight reflecting its frequency in the training CNVs. The weighted density in one genomic region is defined as the summation of total weights of duplicons aligned to this region divided by the region length.

Table 3 lists average densities of all CNVs and non-CNV regions separately for each chromosome. The average densities in CNVs and non-CNV regions in the entire genome are 4.307 and 1.767, respectively. The density is significantly higher in CNV than non-CNV regions (*P* < 10^−5^; two-tailed Welch's *t* test). Although our duplicons are annotated from a subset of CNVs in the human genome, the results show that these duplicons also pervasively appear in other known CNV regions. And core duplicons are indeed more common in all CNVs. In non-CNV regions, there could be some CNVs still uncovered, because we still found a few genomic regions with high density.

### 3.5. NAHR Mediated by Flanking Duplicons

**Figure 7:**
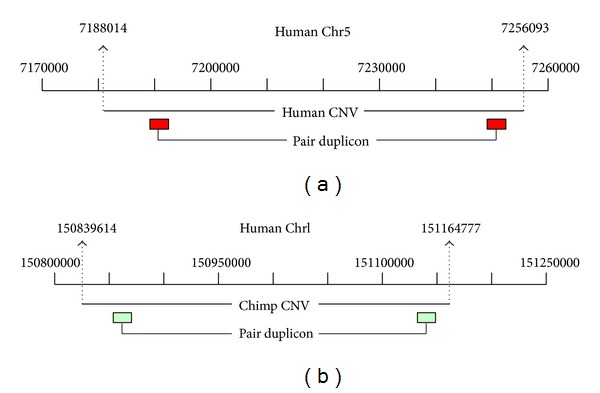
(a) The human CNV is flanked by two identical duplicons at both ends; (b) the chimpanzee CNV is flanked by two identical duplicons at both ends.

A number of studies have noted that genomic regions flanked by duplicated sequences are susceptible to NAHR [1, 9, 15, 21, 22, 29]. These regions are often hotspots of genomic instability that was prone to recurrent CNVs. A recent analysis of a fosmid clone indicated that a CNV is flanked by a pair of duplicons [9]. Figures 7(a) and 7(b) illustrate one human CNV and one chimpanzee CNV with flanking duplicons annotated by our pipeline. As a consequence, we are interested in the distribution of duplicons that locate in flanking regions of CNVs. A pair of duplicons is defined as flanking a CNV if it appears within 25% regions from two ends of the CNV and the similarity (and length) is >90%.

We first investigated 1,097 human CNVs with duplicons annotated by our pipeline [15]. Among them, 1,035 (94%) CNVs have two or more duplicons within their genomic region. 815 out of 1,097 human CNVs (74%) were found to have paired duplicons flanking 25% of both ends. We also analyzed 791 human and chimpanzee CNVs from Perry et al. [17]. Our results indicated that 519 human/chimpanzee CNVs (66%) are also flanked by paired duplicons. Interestingly, each of these CNVs contains averagely ~11 paired duplicons, which could be hotspots of NAHR. This implies that further NAHR occurred within these CNVs may create different breaking points, leading to a complex duplication-within-duplication structure. Thus, these genomic regions may be prone to recurrent CNVs. However, it should be noted that our analysis is based on predefined CNV boundaries, which have been shown to be overestimated [34]. Thus, the requirement of 25% from both ends may eliminate many paired duplicons within real CNV boundaries. Nevertheless, our results provided evidence that there are many paired duplicons within or surrounding a CNV region. As a consequence, boundaries of these complex CNVs may be hard to delineate, since NAHR may reoccur in different breaking points.

### 3.6. Comparison with Duplicon Library

We compared sequences of our duplicons with those in the duplicon library [9], which contains 10,291 duplicon sequences. Our duplicons were BLAST aligned against each duplicon sequence in the library (we considered the alignment results with sequence identities ≥95% and length ≥100 bp). In total, 16,819 (30%) of our duplicons were overlapped with 2,359 (23%) of the duplicon library. It has been shown that ~24% of CNVs are overlapped with SDs [15]. Thus, the difference between our duplicons and duplicon library is probably due to the fact that our duplicons were annotated based on CNVs, whereas duplicons in the library were identified solely based on SDs. However, it should be noted that duplicons with insignificant copy numbers were filtered by our permutation test. Thus, the difference between our duplicons and the duplicon library is not unexpected.

We further compare the distribution of duplicons on chimpanzee specific SDs and CNVs from [17]. These chimpanzee SDs and CNVs are not included in both studies and thus can observe distribution of these duplicons on nonhuman duplications.  Table 4 summarizes the differences between our duplicons and the duplication library. There are 1,048 duplicons in the duplication library overlapped with chimp-specific SDs. Of these, 681 duplicons (65%) are also overlapped with our duplicons. On the other hand, there are 3,310 duplicons annotated by our HMM overlapped with chimp-specific SDs. Of these, 2,554 (82%) are also overlapped with duplicons in the library. In the analysis of CNVs, 1,510 duplicons in the library are located in chimp-specific CNVs. Of these, 886 (59%) duplicons are also overlapped with our duplicons. Among our 2,645 duplicons located within chimp-specific CNVs, 2,209 (84%) are overlapped with duplicons in their library.

These results suggested that duplicons identified by both approaches all appear partially in chimp-specific SDs and CNVs. However, given the higher percentage of our duplicons intersected with both chimp-specific SDs/CNVs and duplicons in the library (82% and 84% versus 65% and 59%), we concluded that duplicons found by our approach are more conservative. This may be due to the requirement of sufficient copy number in our HMM and permutation test, whereas duplicon copies in the library are not validated with a statistical approach.

In terms of efficiency, it is worth mentioning that our HMM is quite efficient compared with the * A*-Bruijn graph algorithm, which requires 29 gigabytes of memory from 32 gigabyte computational cluster [24]. Our HMM can finish the computation within hours on a standard workstation. Consequently, novel duplicons can be efficiently annotated when more CNVs and SDs in other primate genomes are available.

### 3.7. Functional Implication of Duplicons

Our duplicons are smaller subunits within human CNVs. The functional analysis of these duplicons may provide new insight into functional bias not found in previous CNV analysis. We examined the functional bias of our duplicons in gene ontology (GO) categories and compared results with previous analysis of human CNVs. A total of 3,904 genes annotated by Ensembl are overlapped with our duplicons. Tables 5, 6, and 7 list the GO categories (at levels 2 and 3) with over- or underrepresentation of our duplicons (*P* < 0.05; chi-square tests with Bonferroni correction).

For functions related to biological process, we found that eight function categories at level two were significantly biased to our duplicons. At level three, 22 of the 184 GO functions were over- or underrepresented with our duplicons (Table 5). In general, regulation of multicellular organismal process and of biological process is significantly enriched. The highly enriched GO categories overlapped partially with those identified in a previous analysis of CNVs [15], such as regulation of immune system process and regulation of response to stimulus. In contrast to previous analysis, cell adhesion was found to be underrepresented in duplicons. In addition, categories of neurophysiological processes and sensory perception enriched for CNVs are not found to be significantly enriched in duplicons. On the other hand, cell proliferation, oxidation reduction, and metabolic process are found to be significantly underrepresented among duplicons. The impoverishment of these functions probably reflects that purifying selection is against duplicons on dosage of these genes.

In terms of molecular functions, six GO terms at level two and 16 GO terms at level three are over- or underrepresented (Table 6). Specifically, duplicons are overrepresented in catalytic activity, transporter activities, and auxiliary transport protein activity. On the other hand, majority of binding activities, including ion binding, nucleic acid binding, and nucleotide binding are, underrepresented. These results suggest that distinct levels of evolutionary constraint on duplicons vary among functional categories.

## Supplementary Material

The supplementary material includes six phylogenetic trees reconstructed by duplicons within chimpanzee-specific SDs, human-specific SDs, human/chimpamzee-shared SDs, chimpanzee-specific CNVs, human-specific CNVs, and human/chimpamzee-shared CNVs.

## Figures and Tables

**Table 1:** The total number of duplicons of each chromosome.

Chr.	No. of dup.	Chr.	No. of dup.	Chr.	No. of dup.	Chr.	No. of dup.
1	6047	7	5329	13	216	19	2346
2	3607	8	2192	14	889	20	408
3	2142	9	4049	15	2621	21	143
4	1847	10	2039	16	5659	22	1430
5	2537	11	1873	17	3266	X	3078
6	1681	12	1989	18	599	Y	390

**Table 2:** The distribution of duplicons on human/chimpanzee SDs and CNVs. The number of hits stands for the number of SDs/CNVs containing our duplicons. The percentage of hits is shown in brackets. The last column is the average number of duplicons and the percentage of base pair in one SD or CNV.

Data set	Total no.	No. of hits (%)	Average no.
Chimpanzee-specific SDs	219	219 (100%)	43
Human-specific SDs	618	603 (98%)	31
Human/chimp-shared SDs	658	654 (99%)	24

Chimpanzee-specific CNVs	288	288 (100%)	16
Human-specific CNVs	207	206 (99%)	23
Human/chimp-shared CNVs	296	252 (85%)	22

**Table 3:** The average densities of duplicons in CNV and non-CNV regions on each chromosome.

Chr.	CNV	Non-CNV	Chr.	CNV	Non-CNV
1	1.95	1.48	13	1.58	1.51
2	2.13	1.83	14	2.59	1.56
3	2.53	2.11	15	1.80	1.27
4	2.68	2.44	16	1.25	0.92
5	3.03	2.17	17	0.74	0.90
6	2.67	1.93	18	2.51	1.68
7	1.97	1.84	19	0.93	0.44
8	2.77	2.14	20	1.27	1.37
9	2.11	1.50	21	1.50	0.65
10	1.77	1.75	22	0.62	0.22
11	2.90	1.77	X	3.21	3.04
12	1.92	1.97	Y	2.77	0.89

**Table 4:** Comparison of duplicons annotated by HMM and the duplication library. The numbers of (1) duplicons overlapped with each other, (2) duplicons overlapped with chimp-specific SDs, and (3) duplicons overlapped with chimp-specific CNVs are listed for each set of duplicons.

	Our duplicons	Duplib
Total No. of duplicons	56377	10291
No. of duplicons satisfying (1)	16819	2359

No. of duplicons satisfying (2)	3110	1048
No. of duplicons satisfying (1) and (2)	2554	681
Percentage	15% (2554/16819)	29% (681/2359)
Percentage	82% (2554/3110)	65% (681/1048)

No. of duplicons satisfying (3)	2645	1510
No. of duplicons satisfying (1) and (3)	2209	886
Percentage	13% (2209/16819)	38% (886/2359)
Percentage	84% (2209/2645)	59% (886/1510)

**Table 5:** GO analysis of biological process at levels 2 and 3. *P* values are computed by chi-square test with Bonferroni correction.

GO term	GO category	* P* value	Obs./exp.
Level 2			
GO:0000003	Metabolic process	4.36 × 10^−9^	0.75
GO:0001906	Multicellular organismal process	8.22 × 10^−8^	1.36
GO:0002376	Biological adhesion	1.10 × 10^−6^	0.28
GO:0008152	Cellular process	3.53 × 10^−6^	1.16
GO:0009987	Developmental process	4.35 × 10^−6^	1.33
GO:0010926	Positive regulation of biological process	4.70 × 10^−3^	1.38
GO:0016032	Regulation of biological process	1.90 × 10^−2^	0.86
GO:0022414	Locomotion	2.80 × 10^−2^	0.44

Level 3			
GO:0048856	Anatomical structure development	1.40 × 10^−13^	1.71
GO:0051239	Regulation of multicellular organismal process	6.28 × 10^−13^	2.33
GO:0043170	Macromolecule metabolic process	1.53 × 10^−9^	0.65
GO:0009058	Biosynthetic process	2.64 × 10^−9^	0.57
GO:0002682	Regulation of immune system process	1.10 × 10^−8^	2.70
GO:0019222	Regulation of metabolic process	1.74 × 10^−8^	0.53
GO:0007275	Multicellular organismal development	8.82 × 10^−8^	1.53
GO:0048518	Positive regulation of biological process	5.32 × 10^−7^	1.68
GO:0007154	Cell communication	4.69 × 10^−6^	0.65
GO:0001816	Cytokine production	4.98 × 10^−6^	2.89
GO:0051656	Establishment of organelle localization	6.84 × 10^−6^	4.29
GO:0045321	Leukocyte activation	1.35 × 10^−5^	2.44
GO:0032879	Regulation of localization	3.90 × 10^−5^	2.14
GO:0044238	Primary metabolic process	1.62 × 10^−4^	0.77
GO:0001775	Cell activation	1.92 × 10^−4^	2.21
GO:0055114	Oxidation reduction	2.17 × 10^−4^	0.15
GO:0048583	Regulation of response to stimulus	5.14 × 10^−4^	2.24
GO:0051050	Positive regulation of transport	6.46 × 10^−4^	2.84
GO:0007155	Cell adhesion	1.08 × 10^−3^	0.34
GO:0032898	Neurotrophin production	6.88 × 10^−3^	18.9
GO:0060033	Anatomical structure regression	1.81 × 10^−2^	9.47
GO:0008283	Cell proliferation	2.39 × 10^−2^	0.45

**Table 6:** GO analysis of molecular function at levels 2 and 3. *P* values are computed by chi-square test with Bonferroni correction.

GO term	GO category	* P* value	Obs./exp.
Level 2			
GO:0003824	Catalytic activity	1.53 × 10^−33^	1.78
GO:0005488	Binding	1.41 × 10^−27^	0.62
GO:0005215	Transporter activity	4.72 × 10^−14^	2.08
GO:0030528	Transcription regulator activity	3.31 × 10^−5^	0.37
GO:0015457	Auxiliary transport protein activity	4.18 × 10^−3^	3.92
GO:0005198	Structural molecule activity	1.01 × 10^−2^	0.40

Level 3			
GO:0022857	Transmembrane transporter activity	7.32 × 10^−31^	3.10
GO:0004133	Glycogen debranching enzyme activity	4.35 × 10^−30^	71.2
GO:0016740	Transferase activity	4.10 × 10^−28^	2.52
GO:0022892	Substrate-specific transporter activity	1.64 × 10^−25^	2.83
GO:0043167	Ion binding	3.89 × 10^−24^	0.12
GO:0003676	Nucleic acid binding	1.42 × 10^−14^	0.23
GO:0000166	Nucleotide binding	6.41 × 10^−11^	0.15
GO:0016491	Oxidoreductase activity	8.19 × 10^−9^	2.31
GO:0005515	Protein binding	2.84 × 10^−6^	0.67
GO:0016787	Hydrolase activity	3.77 × 10^−6^	1.61
GO:0016787	Transcription factor activity	1.97 × 10^−5^	0.07
GO:0016787	Channel regulator activity	9.61 × 10^−4^	4.97
GO:0016787	Bacterial binding	9.69 × 10^−4^	8.21
GO:0016787	Cell surface binding	3.80 × 10^−3^	5.93
GO:0016787	Peptide binding	4.77 × 10^−2^	1.90
GO:0016787	Signal transducer activity	9.11 × 10^−2^	1.35

**Table 7:** GO analysis of cellular component at levels 2 and 3. *P* values are computed by chi-square test with Bonferroni correction.

GO term	GO category	* P* value	Obs./exp.
Level 2			
GO:0032991	Macromolecular complex	4.14 × 10^−15^	1.97
GO:0044422	Organelle part	8.08 × 10^−9^	1.59
GO:0005576	Extracellular region	3.09 × 10^−5^	0.35

Level 3			
GO:0043234	Protein complex	3.42 × 10^−20^	2.36
GO:0044422	Organelle part	2.22 × 10^−9^	1.65
GO:0044446	Intracellular organelle part	5.95 × 10^−9^	1.64
GO:0044463	Cell projection part	1.17 × 10^−8^	5.17
GO:0042995	Cell projection	2.09 × 10^−5^	2.48
GO:0016020	Membrane	8.17 × 10^−5^	0.65
GO:0044425	Membrane part	8.70 × 10^−5^	0.62
GO:0032311	Angiogenin-PRI complex	5.55 × 10^−4^	21.5
GO:0043227	Membrane-bounded organelle	2.04 × 10^−3^	0.71
GO:0032994	Protein-lipid complex	3.68 × 10^−3^	7.18
GO:0034358	Plasma lipoprotein particle	3.68 × 10^−3^	7.18
