# Supplementary material for: A Novel Framework for the Identification and Analysis of Duplicons between Human and Chimpanzee
Source: Biomed Res Int. 2013 Aug 1;2013:264532. doi: 10.1155/2013/264532 (PMC3747353; doi:10.1155/2013/264532)
Supplement: Supplementary file 1 — The supplementary material includes six phylogenetic trees reconstructed by duplicons within chimpanzee-specific SDs, human-specific SDs, human/chimpamzee-shared SDs, chimpanzee-specific CNVs, human-specific CNVs, and human/chimpamzee-shared CNVs. [file 264532.f1.pdf]

Supplementary Figures

In the supplementary material, we illustrate six phylogenetic trees reconstructed using duplicons within chimpanzee-specific, human-specific, and human/chimpamzee-shared CNVs/SDs.

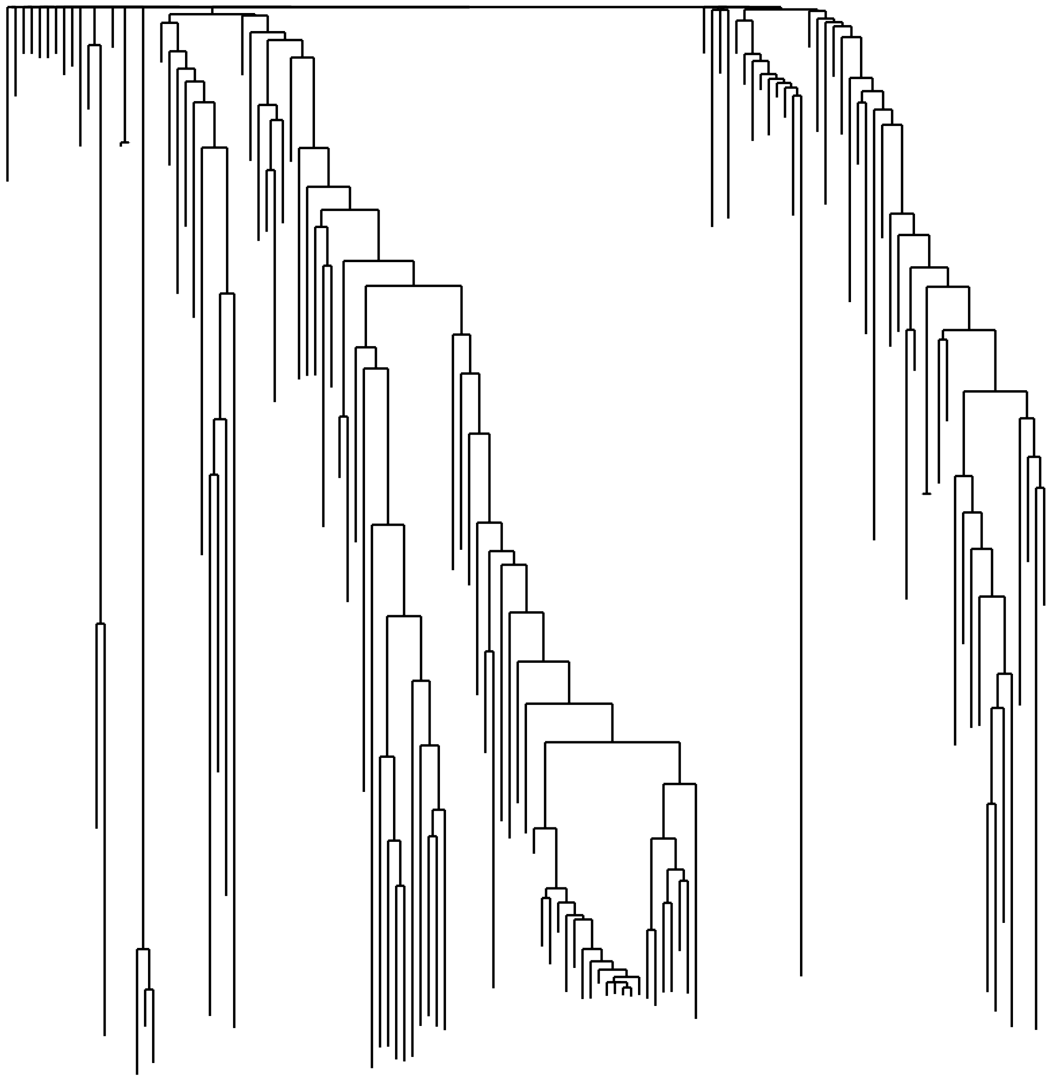

Figure 1: The phylogenetic tree of hierarchical clustering of duplicons for chimpanzee-specific SDs.

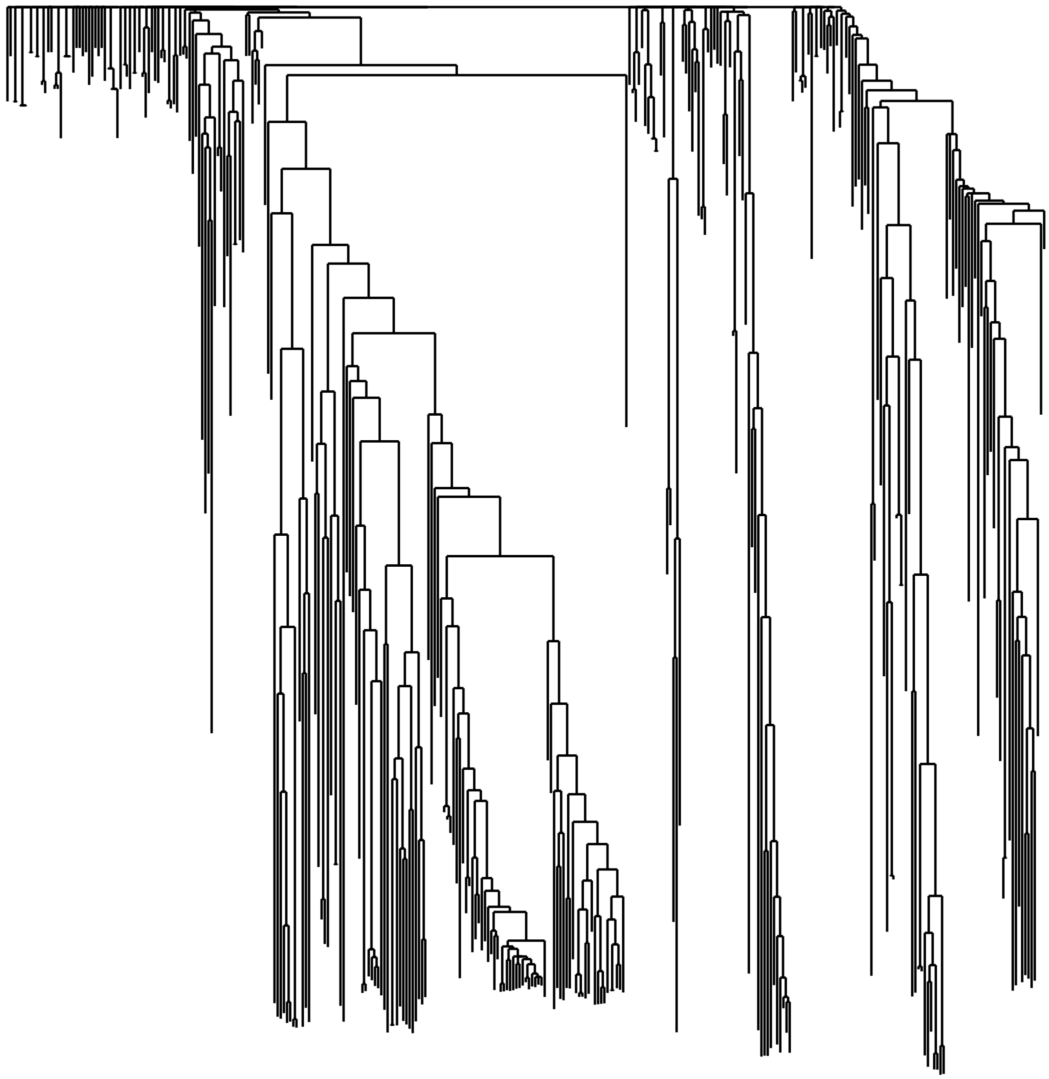

Figure 2: The phylogenetic tree of hierarchical clustering of duplicons for human-specific SDs.

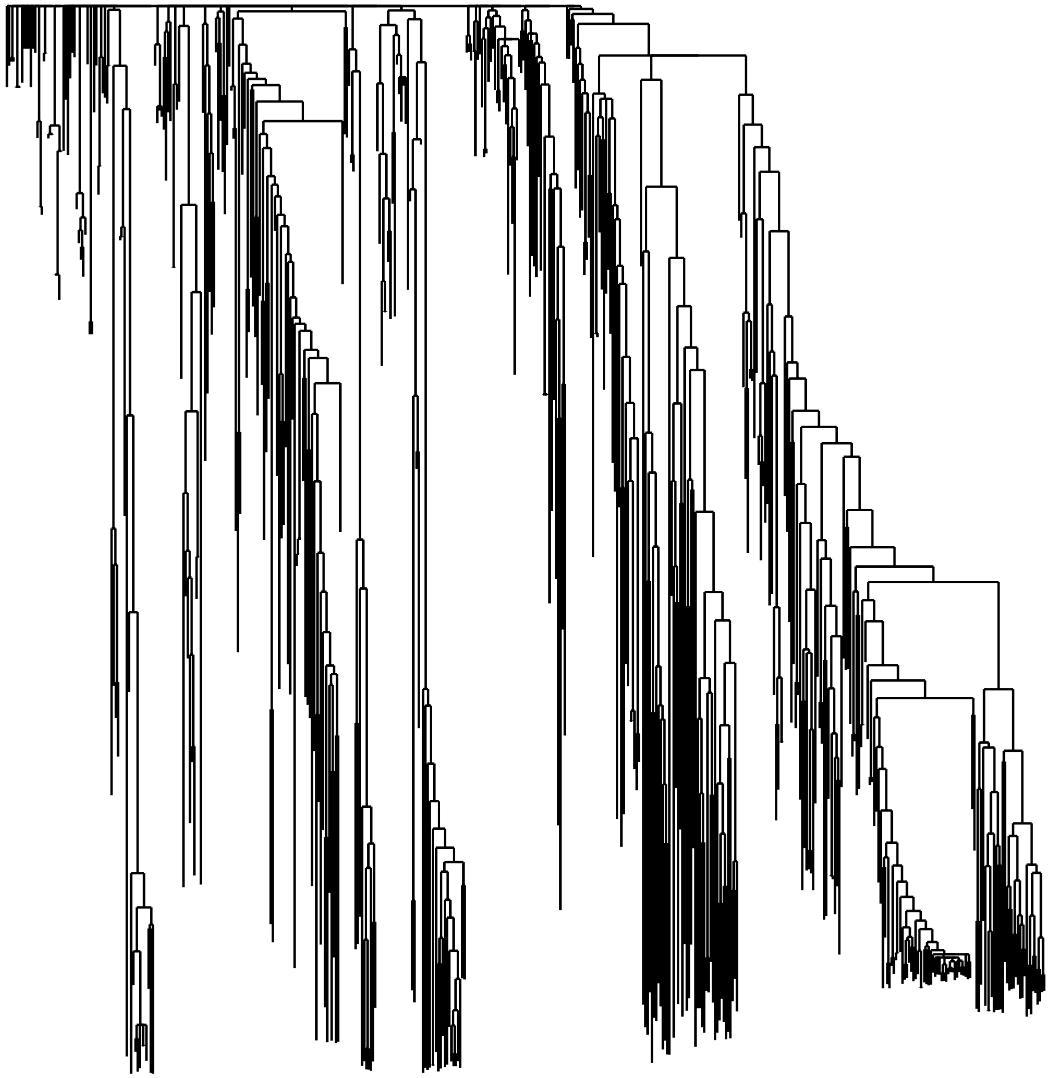

Figure 3: The phylogenetic tree of hierarchical clustering of duplicons for human/chimpanzee-shared SDs.

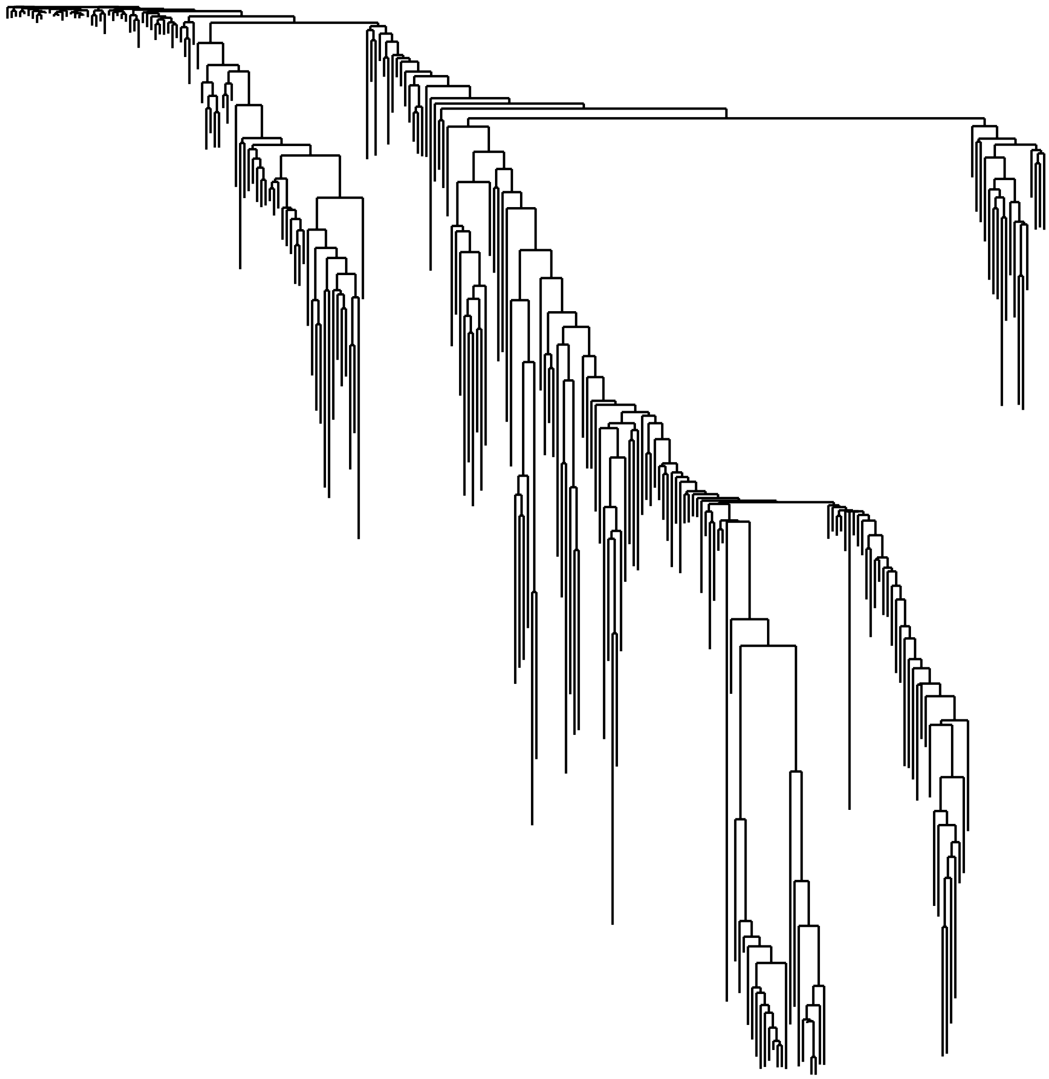

Figure 4: The phylogenetic tree of hierarchical clustering of duplicons for chimpanzee-specific CNVs.

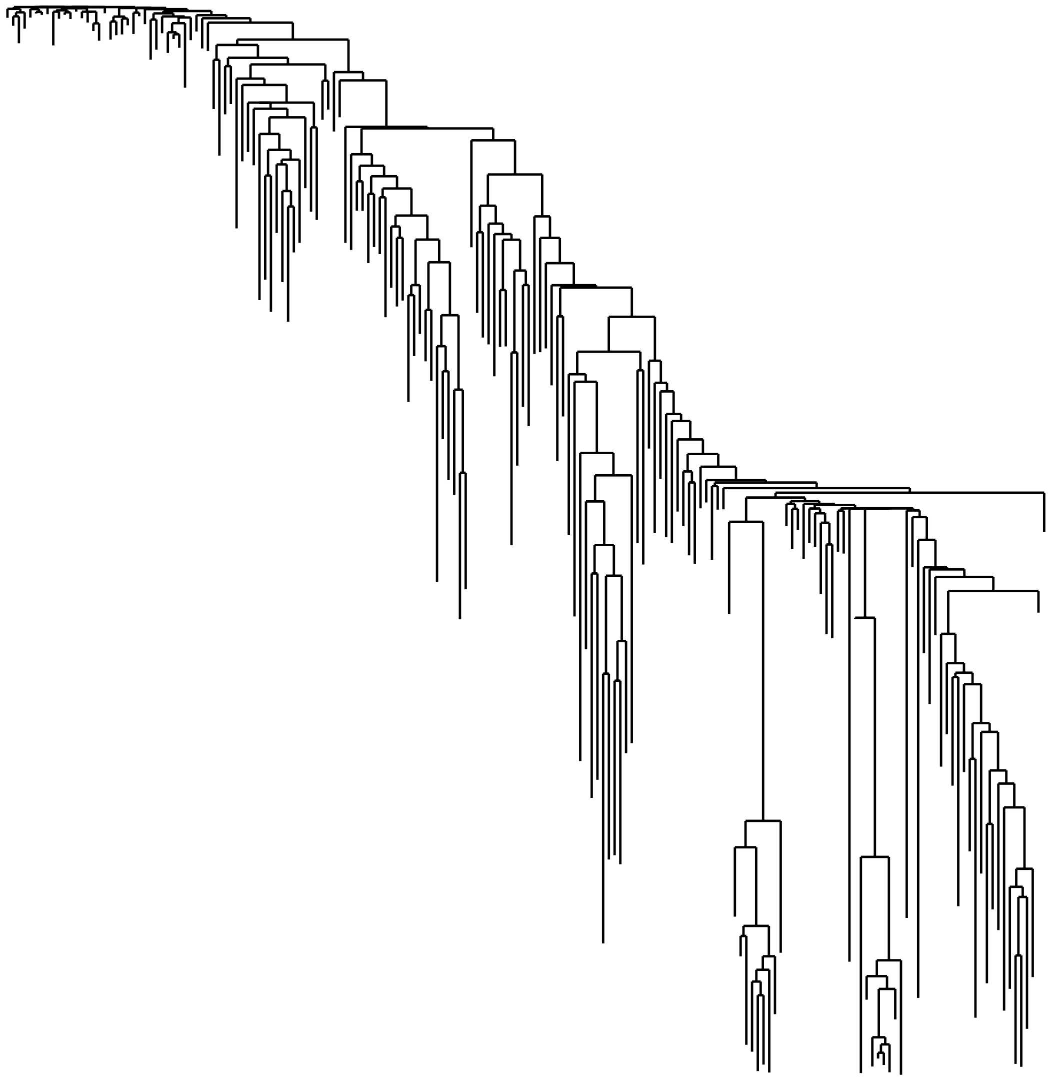

Figure 5: The phylogenetic tree of hierarchical clustering of duplicons for human-specific CNVs.

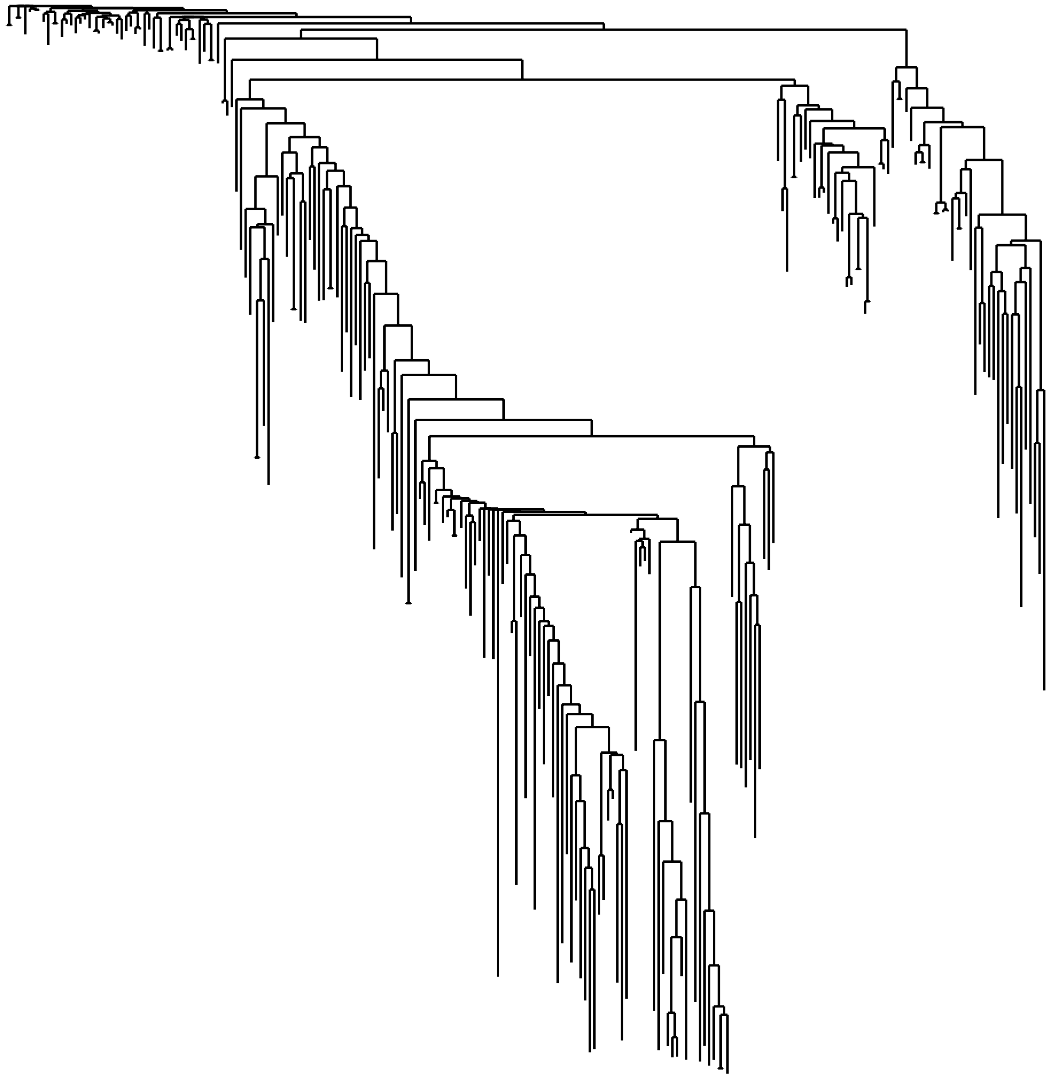

Figure 6: The phylogenetic tree of hierarchical clustering of duplicons for human/chimpanzee-shared CNVs.
